# Multiple Suggested Care Alternatives and Decision-Making of Primary Care Physicians: A Randomized Clinical Trial

**DOI:** 10.1001/jamanetworkopen.2025.42949

**Published:** 2025-11-13

**Authors:** Gemma Altinger, Christopher G. Maher, Caitlin M. P. Jones, Jason Collins, Jeffrey A. Linder, Katy J. L. Bell, Chung-Wei Christine Lin, Marguerite Tracy, Farzaneh Boroumand, Adrian C. Traeger

**Affiliations:** 1 Institute for Musculoskeletal Health, Sydney Local Health District, Sydney, New South Wales, Australia; 2 Faculty of Medicine and Health, Sydney School of Public Health, University of Sydney, Sydney, New South Wales, Australia; 3 UTS Business School, University of Technology Sydney, Sydney, New South Wales, Australia; 4 Division of General Internal Medicine, Northwestern University Feinberg School of Medicine, Chicago, Illinois; 5 General Practice Clinical School, University of Sydney, Sydney, New South Wales, Australia; 6 School of Mathematical and Physical Sciences, Macquarie University, Sydney, New South Wales, Australia

## Abstract

**Importance:**

Decision support alerts or nudges are used in health care settings to guide clinical decisions toward preferred care. However, it is unclear whether the number of alternatives offered leads to suboptimal decisions.

**Objective:**

To determine the effect of presenting two or more appropriate treatment alternatives, compared to one alternative, on care decisions.

**Design, Setting, and Participants:**

This randomized clinical trial conducted from May 3 to May 8, 2024, included primary care physicians (PCPs) practicing in the US who were recruited from the Qualtrics research network and were randomly assigned 1:1 to review 2 clinical scenarios. Physicians in the control group were presented with 1 appropriate treatment alternative for each scenario and those in the intervention group were presented with 2, 3, or 4 treatment alternatives.

**Intervention:**

Physicians were presented with 2 clinical scenarios—one on surgery referral for hip osteoarthritis and one on opioid prescribing for back pain—and asked to decide whether to remain with an existing management plan or to select an appropriate alternative.

**Main Outcomes and Measures:**

The primary outcome was the proportion of PCPs choosing an alternative over the current management plan. In the secondary analysis, odds ratios (ORs) with 95% CIs were calculated to measure the effect of each additional alternative added to the choice set on decisions in the intervention group (ie, increasing from 2 to 3 to 4).

**Results:**

Among 402 physicians (231 [57.5%] with <10 years of clinical experience), 201 identified as men (50.0%) and were from 46 US states, with 196 (49.5%) from urban or metropolitan areas. Of these, 200 were assigned to the control group (1 alternative across 2 clinical scenarios [400 total treatment decisions]) and 202 to the intervention group (≥2 alternatives across 2 clinical scenarios [404 total treatment decisions]). Physicians in the intervention group had significantly higher odds of choosing an appropriate alternative (251 of 404 treatment decisions [62.1%]) compared with those in the control group (176 of 400 treatment decisions [44.0%]) (adjusted OR, 1.90; 95% CI, 1.09-3.30; *P* = .02). The effect was stronger in the opioid prescribing scenario (30.5% [61 of 200 treatment decisions] vs 56.4% [114 of 202 treatment decisions], OR, 2.95; 95% CI, 1.96-4.45) than the surgery referral scenario (57.5% [115 of 200 treatment decisions] vs 67.8% [137 of 202 treatment decisions], OR, 1.56; 95% CI, 1.04-2.34). Increasing the number of alternatives beyond 2 did not increase the effect.

**Conclusions and Relevance:**

In this randomized clinical trial of 402 PCPs, we found that presenting 2 or more appropriate treatment alternatives increased the odds that physicians would choose an alternative. This challenges earlier suggestions that physicians experience status-quo bias when they are offered more than 1 alternative. Decision support alerts may be more effective when offering multiple appropriate alternatives, rather than only 1.

**Trial Registration:**

ANZCTR Identifier: ACTRN12625001025426

## Key Points

**Question:**

Does offering multiple appropriate treatment alternatives affect the odds of primary care physicians choosing an alternative over the current care plan?

**Findings:**

In this randomized clinical trial of 402 primary care physicians, offering 2 or more appropriate alternatives significantly increased the odds that physicians would choose an alternative (62%) compared with those offered only 1 alternative (44%).

**Meaning:**

Contrary to prior studies suggesting status-quo bias, in this trial, presenting multiple appropriate alternatives in decision support alerts increased the odds that physicians would choose an alternative; indicating that presenting multiple alternatives may improve clinical decision-making and reduce unwarranted variation in health care.

## Teaser

This randomized clinical trial investigates whether providing primary care physicians (PCPs) with 1 vs 2 or more appropriate treatment alternatives in a choice set has an effect on the odds that they would shift from an existing treatment plan to an alternative plan.

## Introduction

Unwarranted health care variation is the inconsistency of clinical care that cannot be explained by patient symptoms or preferences.^1,2^ Health care variation can include overuse of tests and treatments that do not offer any benefit to patients.^3^ It can also include the underuse of care that would benefit patients.^4^ Unwarranted health care variation represents a risk to patient safety and the quality of health care internationally.^5,6^ A common example is the overuse of opioid analgesics and underuse of safer, more effective alternatives, such as nonsteroidal anti-inflammatory drugs (NSAIDs), for patients with low-back pain in primary care.^7,8,9^ Despite efforts to reduce unwarranted health care variation through dissemination of guidelines, public awareness campaigns, and quality improvement projects, the problem persists.^5,10,11,12^

Computerized interventions are increasingly implemented in health care settings to improve care.^13,14^ These interventions can include decision-support tools, best-practice alerts, and suggested alternative nudges.^15,16^ Suggested alternative interventions, in which clinicians are provided a list of treatment alternatives at the point of care, have been used to reduce overuse of antibiotics in primary care^16^ and opioids in emergency departments.^17^ Such interventions may support decision-making by disrupting habitual prescribing patterns, increasing the salience of appropriate alternatives, and signaling which treatment is recommended.^17,18,19^

The effectiveness of these interventions in improving care may depend on the number of appropriate alternatives provided. In a highly cited experiment, Redelmeier and Shafir^20^ suggested that providing 2 care alternatives instead of 1 led physicians to paradoxically remain with an existing management plan. This result was striking because it violated normative choice theory or the Luce Choice Axiom, which states that the introduction of a third alternative should not increase the selection of existing options or the status quo.^21,22^ Redelmeier and Shafir^20^ concluded that providing multiple alternatives creates decision difficulty among physicians, triggering a cognitive bias, known as status-quo bias. The implication was that decision supports should limit the number of options presented to clinicians.

While the study by Redelmeier and Shafir^20^ has been influential in shaping our understanding of clinical decision-making, a more recent study^23^ could not replicate the original findings. Restricting care alternatives shown to clinicians based on limited or outdated evidence could lead to suboptimal care.^24,25,26^ Since the original study, there have been substantial advances in research methods, insights on decision-making, and the influence of electronic health systems in clinical decision-making.^27,28,29,30^ As such, interventions to address unwarranted health care variation require the latest evidence, supported by rigorous scientific methods, to effectively improve health care quality and safety.^31,32^

This randomized clinical trial aimed to determine the effect of the number of appropriate treatment alternatives in a choice set on clinical decision-making. Specifically, we investigated whether providing 2 or more alternatives influenced the odds that primary care physicians (PCPs) would select an alternative treatment or remain with the existing (status-quo) management plan.

## Methods

In this randomized clinical trial, the hypothesis, treatment conditions, and allocation were concealed from participants. After reading the participant information on the online form, participants provided written consent by clicking “yes, I consent.” The Sydney Local Health District Human Ethics Committee approved this trial. The ethics approved study materials are available in the eMethods 1 in Supplement 1, and the trial protocol is provided in Supplement 2. We followed the Consolidated Standards of Reporting Trials (CONSORT) and the Template for Intervention Description and Replication (TIDieR) reporting guidelines.

### Participants

Practicing PCPs in the US who were registered with the Qualtrics research network were invited via email to participate in a survey. Qualtrics is a large online platform that connects researchers with participants. Physicians who completed the clinical scenarios received compensation (equivalent to approximately $22) through their Qualtrics membership.

### Randomization

Physicians were randomly assigned 1:1 to the control or intervention condition (Figure) using computerized randomization via the Qualtrics platform. Participant flow and randomization were programmed by trial investigators with support from a Qualtrics data manager. Investigators were blinded to the group allocation and responses until the sampling was complete, and the survey was closed. To ensure data integrity, the independent data manager removed responses that did not meet their data-quality standards (eg, rapid completion time or failed attention checks) before providing the final dataset for analysis.^33^

**Figure.**
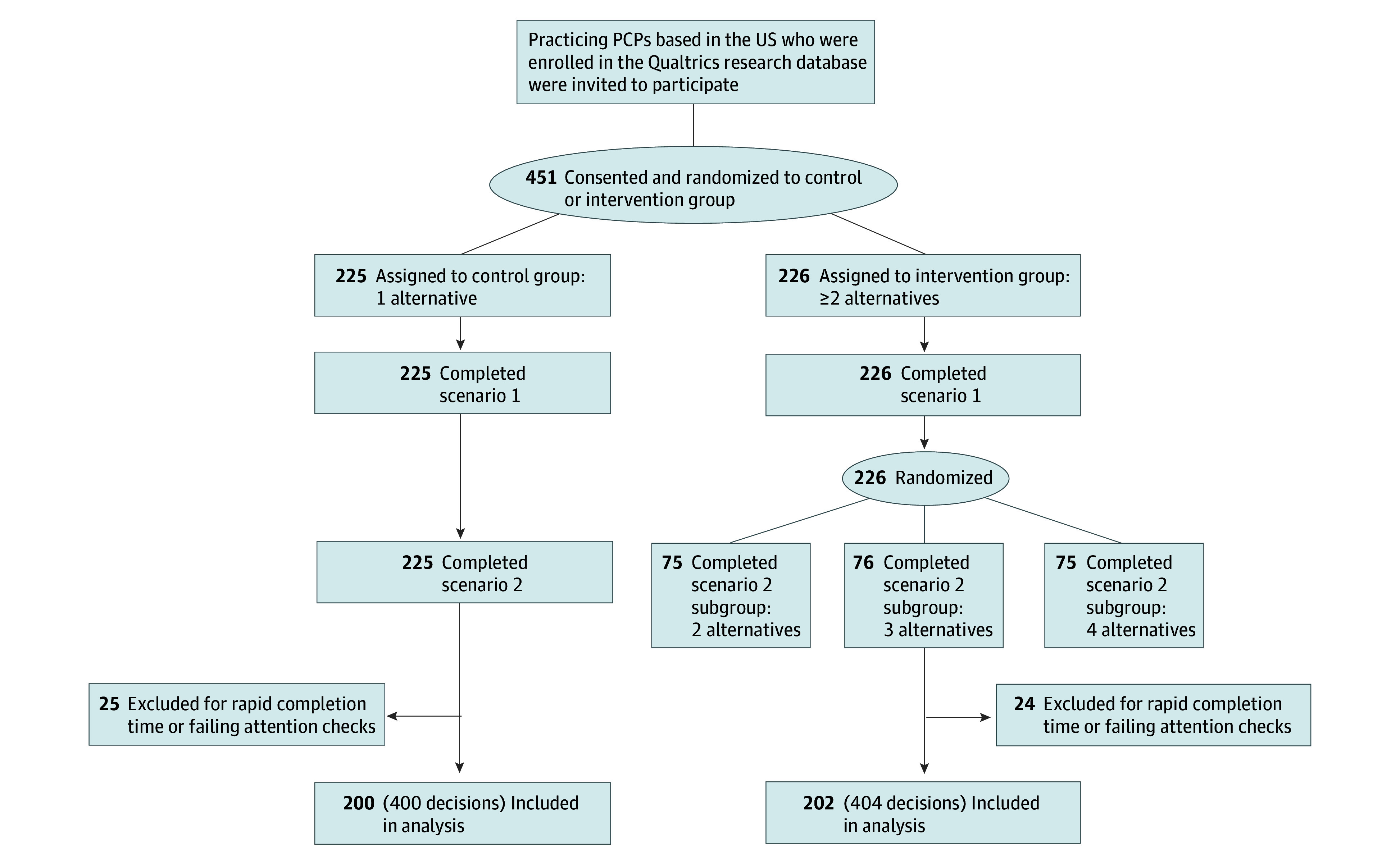
Participant Randomization PCP indicates primary care physician.

### Procedure

Physicians were presented with 2 clinical scenarios commonly seen in primary care. Each scenario—one about a surgery referral for hip osteoarthritis, and the other about opioid prescribing for lower-back pain—involved a decision about whether to remain with an existing management plan or to select an alternate plan. In the control condition, physicians received 1 appropriate treatment alternative. In the intervention condition, physicians could receive 2, 3, or 4 appropriate alternatives.

### Interventions

The clinical scenarios were designed during protocol development (Supplement 2) with the research team that included PCPs, behavioral economists, and investigators with expertise in research methods. Guidance on designing experimental vignette studies to identify factors of health care variation was followed.^34^ The scenarios were carefully designed to assess whether the number of treatment alternatives influenced care decisions.^35,36,37^

Key design elements were implemented. First, the scenarios and treatment alternatives were designed to have comparable trade-off complexity.^35^ The scenarios included details about the patient and clinical situation to ensure that choosing to remain with the current care plan or an alternative could both be considered reasonable clinical decisions. Treatment alternatives were limited to NSAIDs with similar risk-benefit trade-offs and dose frequency to ensure no option was obviously superior or inferior. As such, other guideline concordant care options, such as exercise, were not included. This allowed us to isolate the effect of the number of alternatives on decision-making, rather than measuring physician preferences for specific treatments. Second, to further control for physician preferences, alternatives were randomly selected from a longer list of appropriate NSAID alternatives for each participant (eMethods 1 in Supplement 1). Third, physicians were asked to assume there was no difference in financial costs for any option and there was no single right answer. Scenarios and treatment alternatives were considered credible, relevant, and clear by clinician investigators.

Scenario 1 featured a patient with chronic hip pain and osteoarthritis and replicated the Redelmeier and Shafir^20^ study, with updates to ensure clinical relevance in 2024 (eg, drug names, patient’s profession). In this scenario the patient had been seeing a physiotherapist and walked daily. They had tried 1 NSAID but stopped due to limited efficacy. The existing management plan (or status-quo option) in this scenario was to refer the patient to an orthopedic surgeon, without trying a new NSAID. The alternative options presented were to continue with the referral and also start the patient on a new NSAID. Control physicians were presented with 1 NSAID alternative and intervention physicians were presented with 2 NSAID alternatives (eTable 1 in Supplement 1).

Scenario 2 featured a patient with chronic low-back pain. In this scenario the patient had been managing their back pain with physiotherapy and regular exercise over the past few months. Approximately 2 weeks ago the patient received a 3-day supply of an opioid analgesic (oxycodone) to help manage a flare up. In this scenario the patient had requested another 3-day supply of oxycodone but, when the physician initiated the order, an alert was triggered, suggesting that they consider an NSAID instead. The status-quo option was to continue with the opioid analgesic, and the alternative was to try an NSAID. Control physicians were presented with 1 NSAID alternative and intervention physicians were presented with 2, 3, or 4 NSAID alternatives (eTable 2 in Supplement 1).

### Outcomes

The primary outcome was the proportion of PCPs who chose an alternative treatment option. The secondary outcome was the effect of increasing the number of alternatives to 3 and 4 alternatives on clinical decision-making. Baseline characteristics were collected after physicians completed the clinical scenarios (Table 1).

**Table 1.** Participant Characteristics

Characteristic	Participants, No. (%)		
	All (n = 402)	Control group	Intervention group
		1 Appropriate treatment alternative (n = 200)	≥2 Appropriate treatment alternatives (n = 202)
Gender			
Women	199 (49.5)	116 (58.0)	83 (41.1)
Men	201 (50.0)	84 (42.0)	117 (57.9)
Prefer not to say	2 (0.5)	0	2 (1.0)
Years in clinical practice			
<5	88 (21.9)	39 (19.5)	49 (24.3)
5-9	143 (35.6)	67 (33.5)	76 (37.6)
10-14	98 (24.4)	60 (30.0)	38 (18.8)
15-19	49 (12.2)	25 (12.5)	24 (11.9)
≥20	24 (6.0)	9 (4.5)	15 (7.4)
Proportion of working hours in clinical practice, %			
≤25	68 (16.9)	34 (17.0)	34 (16.8)
26-50	132 (32.8)	68 (34.0)	64 (31.7)
51-75	155 (38.6)	81 (40.5)	74 (36.6)
>75	47 (11.9)	17 (8.5)	30 (14.9)
Time instructing medical students, h/wk			
≤5	116 (28.9)	47 (23.5)	69 (34.2)
6-10	164 (40.8)	81 (40.5)	83 (41.1)
11-15	95 (23.6)	61 (30.5)	34 (16.8)
≥16	27 (6.7)	11 (5.5)	16 (7.9)
Rurality			
Rural	52 (12.9)	28 (14.0)	24 (11.9)
Suburban	154 (38.3)	76 (38.0)	78 (38.6)
Urban or metropolitan	196 (48.8)	96 (48.0)	100 (49.5)

### Statistical Analysis

The study by Redelmeier and Shafir^20^ observed an absolute difference of 19% between groups. To detect a 14% difference with 80% power and a 2-sided α = .05, we required 198 physicians per condition completing 2 scenarios. Our primary analysis used a binary logistic regression interaction model with generalized estimation equations (GEE) to estimate the effect of the intervention on the proportion of clinicians choosing an alternative (eMethods 1 in Supplement 1). Our GEE analysis accounted for within participant clustering (as each physician completed 2 scenarios) and interaction effects between the group and the clinical scenario (eMethods 2 in Supplement 1). Effect sizes were presented as odds ratios (OR) with 95% CIs. A 2-sided *P* < .05 was considered statistically significant. As a secondary exploratory analysis, we estimated unadjusted effects in the total sample and in each scenario using 3 univariate logistic regression models. We also calculated the number and proportion choosing an alternative in the subgroups presented with 2, 3, or 4 alternatives. Statistical analyses were conducted using R, version 4.3.2 (R Project for Statistical Computing).

## Results

Of the 402 physicians (231 [57.5%] with <10 years of clinical experience; 171 [42.5%] with ≥10 years clinical experience) included in the analyses, 199 identified as women (49.5%), 201 identified as men (50.0%) and 2 preferred not to say (0.5%). Participants were from 46 US states, with 196 (49.0%) from urban or metropolitan areas (Table 1). A total of 451 physician participants were randomized and completed the scenarios; however, 49 responses (10.9%) had rapid completion time or failed data integrity checks and were excluded (Figure). Of these participants, 200 physicians (with 400 decisions in total) were assigned to the control group and 202 (with 404 decisions in total) were assigned to the intervention group. Physicians completed the scenarios between May 3 and 8, 2024.

### Primary Outcome

Physicians had significantly higher odds of choosing a treatment alternative when presented with 2 or more appropriate alternatives rather than just 1 (44.0% [176 of 400 treatment decisions] in control vs 62.1% [251 of 404 treatment decisions] in intervention; unadjusted OR, 2.09; 95% CI, 1.58-2.77) (Table 2). The GEE model, which accounted for both clustering and interaction effects, estimated an adjusted OR of 1.90 (95% CI, 1.09-3.30; *P* = .02), indicating a statistically significant effect of the intervention. The intervention had a larger effect in the opioid prescribing scenario (30.5% [61 of 200 treatment decisions] vs 56.4% [114 of 202 treatment decisions]; unadjusted OR, 2.95; 95% CI, 1.96-4.45) compared with the surgical referral scenario (57.5% [115 of 200 treatment decisions] vs 67.8% [137 of 202 treatment decisions], unadjusted OR, 1.56; 95% CI, 1.04-2.34).

**Table 2.** Effects of the Number of Appropriate Alternatives on Choice of an Alternative

Variable	No./total No. of decisions (%)		Effect size, OR (95% CI)	
	1 Treatment alternative (n = 200)^a^	≥2 Treatment alternatives (n = 202)^a^	Unadjusted^b^	Adjusted^c^
Total decisions	400	404	NA	NA
Primary outcome: % choosing an alternative	176/400 (44.0)	251/404 (62.1)	2.09 (1.58-2.77)	1.90 (1.09-3.30)^d^
Scenario 1: Surgical referral with or without NSAIDs	115/200 (58.5)	137/202 (67.8)	1.56 (1.04-2.34)	NA
Scenario 2: Opioid or NSAIDs	61/200 (30.5)	114/202 (56.4)	2.95 (1.96-4.45)	NA

Abbreviations: NA, not applicable; NSAID, nonsteroidal anti-inflammatory drug; OR, odds ratio.

^a^ The control group had 1 appropriate treatment alternative and the intervention group had 2 or more appropriate treatment alternatives.

^b^ Estimate from a univariate logistic regression model. Does not account for within participant clustering.

^c^ Adjusted for within participant clustering and includes interaction effects.

^d^ *P* = .02.

### Secondary Outcome

Our exploratory analysis found that increasing the number of appropriate alternatives beyond 2 did not increase the odds that physicians would choose an alternative. Physicians who were randomized to receive 2, 3, and 4 alternatives in the opioid scenario had similar proportions choosing an alternative (55.2% [37 of 67], 58.8% [40 of 68], and 55.2% [37 of 67] respectively) compared with 30.5% (61 of 200) choosing the alternative in the control condition (Table 3).

**Table 3.** Effects of the Number of Appropriate Alternatives on Choice of an Alternative in Each Scenario Between Subgroups

Variable	No./total No. of decisions (%)			
	Control group	Intervention group		
	1 Treatment alternative (n = 200)^a^	2 Treatment alternatives (n = 67)	3 Treatment alternatives (n = 68)	4 Treatment alternatives (n = 67)
No. of decisions	400	269	68	67
Primary outcome in both scenarios: % choosing an alternative	176/400 (44.0)	174/269 (64.6)	40/68 (58.8)	37/67 (55.2)
Scenario 1: Surgical referral with or without NSAIDs	115/200 (57.5)	137/202 (67.8)	NA	NA
Scenario 2: Opioid or NSAIDs	61/200 (30.5)	37/67 (55.2)	40/68 (58.8)	37/67 (55.2)

Abbreviations: NA, not applicable; NSAID, nonsteroidal anti-inflammatory drug.

^a^ The control group had the option to select 1 appropriate treatment alternative.

## Discussion

The aim of this study was to better understand clinical decisions to improve future health system design. Our findings challenge suggestions that physicians experience cognitive bias in medical decisions that offer multiple alternatives. Rather than causing physicians to remain with the existing management plan, providing multiple alternatives substantially increased the odds that participants would shift away from an existing management plan to an appropriate alternative. Presenting 2 or more appropriate alternatives had larger effects in the scenario about opioids than in the scenario about surgery. These findings were independent of clinician preferences and robust to attention checks.

Our results are unlikely to be explained by preferences or by physicians randomly selecting an option. To control for clinician preferences, we selected alternatives with similar risk-benefit trade-offs. The specific alternatives presented to each physician were randomized from a longer list of appropriate alternatives, minimizing the influence of individual preferences on our outcome data. To reduce the risk of participants rushing through and randomly selecting options, we excluded data from those who completed the experiment too fast, or who failed attention checks. Participants who were offered multiple appropriate alternatives by design had a higher probability of selecting an alternative from their decision set. However, we found no evidence that increasing the number of appropriate alternatives offered beyond 2 increased the odds of choosing an alternative.

According to Redelmeier and Shafir,^20^ introducing an additional treatment alternative increased the proportion of physicians remaining with the current care plan, rather than choosing an alternative (47% choosing an alternative in the control vs 28% choosing an alternative in the intervention; *P* < .001). Redelmeier and Shafir^20^ suggested that this finding was attributable to status-quo bias, where they paradoxically remained with the existing management plan, potentially due to the increased decision difficulty when an extra treatment option was added. Chernev et al^35^ described this difficulty as a response to experiencing choice overload. However, we found the opposite. Offering 2 or more alternatives significantly increased the odds that physicians would shift away from an existing management plan and choose an appropriate alternative.

This randomized clinical trial found no evidence of status-quo bias when physicians were presented with multiple choice alternatives. While our tightly controlled design allowed us to robustly test for status-quo bias, it did not permit examination of other decision-making strategies. We propose possible explanations that may inform future research. Prior evidence suggests that experts manage complexity by organizing information into meaningful subgroups or chunks.^38,39^ In this trial, physicians may have mentally grouped the treatment options into clinically meaningful subgroups to simplify decision-making. Given that the risks and benefits were balanced across options, physicians may have categorized treatments into NSAIDs and non-NSAID subgroups. The value share or proportion of options belonging to a given subgroup may have influenced decision-making by increasing salience of options and potentially conveying implicit recommendations.^27^ Tannenbaum and colleagues,^27^ for example, found that the presentation of choice sets influenced prescribing behavior. They found when aggressive treatments were grouped together on a single line, and preferred treatments were listed individually, PCPs were less likely to choose aggressive options by 11 percentage points. Although our trial did not explicitly group options into subgroups, the number of options may have influenced how physicians cognitively organized and assessed them. These results are consistent with evidence that suggests choice behavior is adaptive and sensitive to the framing and structure of decisions.^28,30,40^

### Implications for Research, Policy, and Practice

Redelmeier and Shafir^20^ suggested that physicians were less likely to choose an alternative when offered 2 treatment alternatives, rather than just 1, concluding this was evidence of status-quo bias. Our findings challenge this suggestion. According to our randomized clinical trial, restricting the number of appropriate alternatives offered to physicians may lead to suboptimal decision-making. Alternatively, decision support interventions that offer multiple preferred alternatives in a choice set may encourage higher-quality care and reduce unwarranted health care variation.

Our trial highlights the need to critically reexamine early studies of cognitive bias in clinical decision-making. The introduction of electronic health systems and telehealth means physicians are exposed to more information than previous generations, further pointing to the need for updated evidence that better reflects contemporary clinical decision-making.^41^ Future trials should evaluate the effectiveness of offering 2 or more appropriate alternatives, such as in a best practice alert or suggested alternative nudge, on decision-making in a clinical setting. The effect of introducing more treatment subgroups, such as pharmacological and nonpharmacological treatments, should also be examined. To ensure interventions improve care, they should be evaluated through randomized clinical trials or randomized pilot testing, with attention given to changes in clinical care, unintended consequences, and patient outcomes.^42,43^

### Strengths and Limitations

This trial has several strengths that may help explain why our results differ from those of previous studies. First, we introduced a second primary care scenario where each treatment alternative was independent and evenly matched in terms of risks and benefits. In contrast, the study by Redelmeier and Shafir^20^ examined only 1 primary care scenario on osteoarthritis, and all treatment options included a referral for surgery, which could have confounded the results. Our results may differ due to the rigor of our design, an increased familiarity with best practice care for patients with hip osteoarthritis, or both.^44^ Additionally, the treatment alternatives in the present trial were specific in terms of dose, frequency, and route, reducing the influence of physicians’ prior experience, knowledge, or preferences. Second, our second scenario incorporated a best practice suggested alternative alert, increasing the relevance of our trial and better reflecting how clinicians are likely to be presented with multiple alternatives in real clinical practice.^16,17,45^ Third, by using an online platform, we were able to recruit a more geographically diverse sample of practicing PCPs. Our physician participants were from 46 states across the US, including metropolitan, suburban, and rural locations, unlike previous studies that sampled only academic physicians in one specific state or province.^20,46^ Finally, unlike the study by Redelmeier and Shafir^20^, this trial was preregistered and adhered to the Template for Intervention Description and Replication (TIDieR) reporting guideline.^47,48^

Despite addressing several key limitations of prior studies, this trial has limitations. First, online studies cannot fully replicate the complexity of real clinical decision-making where guideline-concordant treatment options may be more varied and multifaceted. However, experiments in controlled conditions, including survey vignette studies, can be an effective, acceptable method for replication and identifying factors that play a role in health care variation.^34,49^ This approach allowed us to isolate the effect of multiple alternatives on decision-making, minimize confounding variables and avoid harm to patients that could arise from testing the hypothesis in clinical settings.^49^ Second, both of our scenarios focus on care for musculoskeletal conditions, which may limit the generalizability of our findings to other clinical areas. This is also the case for previous studies. Finally, 49 participants were excluded after randomization due to rapid completion or failed attention checks. These data integrity procedures help ensure that responses are not provided by participants who are not meaningfully engaging with the scenarios.^33^ Because Qualtrics only provided data for participants who passed these checks, we were unable to determine how many physicians were originally invited or to compare characteristics of those who were included vs excluded. As with all voluntary studies, our findings may also be subject to nonresponse bias if physicians who participated differ systematically from those who did not.^50^ Nevertheless, our sample is likely to be more representative than other studies that surveyed only academic physicians in a single city and required them to return responses by mail.

## Conclusions

The findings of this randomized clinical trial challenge the suggestion that physicians experience cognitive bias in medical decision-making situations that offer multiple treatment alternatives. We found that offering 2 or more alternatives increased the likelihood that physicians would opt for a preferred treatment option. Interventions that offer multiple preferred alternatives may better support clinicians to deliver higher-quality care.

## Notes

Supplement 1. **eMethods 1.** Ethics Approved Study Materials **eTable 1.** Scenario 1 – Surgery Referral Scenario and Randomized Treatment Alternatives Present to Control and Intervention Groups **eTable 2.** Scenario 2 – Opioid Prescribing Scenario and Randomized Treatment Alternatives Presented to Control and Intervention Groups **eMethods 2.** GEE Model

Supplement 2.
Trial Protocol

Supplement 3.
Data Sharing Statement
