# Supplementary material for: Multiple Suggested Care Alternatives and Decision-Making of Primary Care Physicians: A Randomized Clinical Trial
Source: JAMA Netw Open. 2025 Nov 13;8(11):e2542949. doi: 10.1001/jamanetworkopen.2025.42949 (PMC12616461; doi:10.1001/jamanetworkopen.2025.42949)
Supplement: Supplement 1. — eMethods 1. Ethics Approved Study Materials eTable 1. Scenario 1 – Surgery Referral Scenario and Randomized Treatment Alternatives Present to Control and Intervention Groups eTable 2. Scenario 2 – Opioid Prescribing Scenario and Randomized Treatment Alternatives Presented to Control and Intervention Groups eMethods 2. GEE Model [file jamanetwopen-e2542949-s001.pdf]

Supplemental Online Content

Altinger G, Maher CG, Jones CMP, et al. Multiple suggested care alternatives and decision-making of primary care physicians: a randomized clinical trial. *JAMA Netw Open*. 2025;8(11):e2542949. doi:10.1001/jamanetworkopen.2025.42949

eMethods 1. Ethics Approved Study Materials

eTable 1. Scenario 1 – Surgery Referral Scenario and Randomized Treatment Alternatives Presented to Control and Intervention Groups

eTable 2. Scenario 2 – Opioid Prescribing Scenario and Randomized Treatment Alternatives Presented to Control and Intervention Groups

eMethods 2. GEE Model

This supplemental material has been provided by the authors to give readers additional information about their work.

© 2025 Altinger G et al. *JAMA Network Open*

eMethods 1. Ethics study materials

Participant invitation

Subject: You are invited to complete a short survey to help us understanding clinical decision making to improve future health system design

You are invited to participate in a 5-minute survey to help us improve future health system design. If you consent you will be randomised into one of two groups. We will then ask you to respond to 2 clinical scenarios that may be commonly seen in primary care, these have no right or wrong answers. Each scenario should take no longer than 1-2 minutes to complete. We will then ask you a few background questions about your location of practice, clinical and teaching experience. Your responses will be confidential and anonymous.

Thank you for contributing to this study.

Adrian Traeger | PhD
USYD Robinson Fellow
The University of Sydney, Gadigal Country
Faculty of Medicine and Health, School of Public Health, **Institute for Musculoskeletal Health**

Level 10N, King George V Building, Royal Prince Alfred Hospital (C39)
PO Box M179, Missenden Road, NSW, 2050
Tel +61 416 122 784

adrian.traeger@sydney.edu.au | **sydney.edu.au/adrian.traeger** | **@adrian_traeger** |

© 2025 Altinger G et al. *JAMA Network Open*

Understanding clinical decision making to improve future health system design: a randomised experiment

PARTICIPANT INFORMATION SHEET

**Short Title**	Understanding clinical decisions
**Project Sponsor**	University of Sydney, Australia
**Coordinating Principal Investigator/ Principal Investigator**	Dr Adrian Traeger
**Location**	Online

1. Introduction

You are invited to take part in a research study looking at better understanding clinical decisions to improve future health system design. You are eligible to participate because you are a primary care physician registered with Qualtrics, practicing in the United States. This study will present participants two clinical scenarios and participants will be asked to select a treatment option.

The study is being conducted within the Institute of Musculoskeletal Health, a partnership between the University of Sydney and The Sydney Local Health District. by Dr Adrian Traeger, Senior Researcher at the University of Sydney, Australia. Ms Gemma Altinger is conducting the study to partially fulfil the requirements of Master of Philosophy at the University of Sydney under the supervision of Dr Traeger. The study is being sponsored by the University of Sydney, Australia. The study is being supported by a research grant from the Faculty of Medicine and Health, The University of Sydney (CIA Traeger).

© 2025 Altinger G et al. *JAMA Network Open*

This Participant Information Sheet (PIS) will tell you what is involved in the study and help you decide whether or not you wish to take part. Please read this information carefully. If there is anything you do not understand or if you feel you need more information about anything, please ask.

2. Study Procedures

If you agree to participate in this study, you will be asked to sign the Participant Consent Form at the end of this document. You will then be randomised into one of two groups and asked to read two clinical scenarios and answer a single multiple-choice question at the end of each. The two groups will be given different sets of treatment options to choose from. There are no right or wrong answers. You will then be asked to answer 7 simple background questions about your location, years in practice and teaching experience. We expect the survey to take around 5 minutes. If you would like to receive the results of the study, you will have the option to provide your email address at the end – this will be stored in a separate database and cannot be linked to your survey responses. Responses will remain anonymous. You can complete this survey on your phone or computer, in a place convenient to you.

3. Risks

There are no foreseeable risks associated with this study.

4. Benefits

While we intend that this research study furthers health system knowledge and may improve health system design in the future, it will not be of direct benefit to you. However, you will be reimbursed for your time as defined by your membership with Qualtrics.

5. Costs

Aside from giving up your time, we do not expect there to be any costs associated.

6. Voluntary Participation

© 2025 Altinger G et al. *JAMA Network Open*

Participation in this study is entirely voluntary. You do not have to take part in it. If you do take part, you can withdraw at any time by closing the online survey. However, only finished surveys will be eligible for financial reimbursement.

Data collected up until the time you withdraw may be included in the study. The study results may be presented at a conference or in a scientific publication. The information you provide for this study will be anonymous. This means that it cannot be re-identified once you have submitted the survey. This means that your information cannot be withdrawn from the study after you submit the survey.

7. Confidentiality

All the information collected from you for the study will be treated confidentially and will be stored on a research database at the University of Sydney. The data will be analysed by the researchers at the University of Sydney. The survey responses will be stored in a study directory on the University of Sydney's server, with access permissible only to the study personnel. A Research Data Management Plan (RDMP) has been created using the Sydney Local Health District RDMP tool. The files will be retained for 10 years from the day the study is completed. Once the retention expires the files will be disposed of. Data will be securely deleted from the RDS once appropriate approval is given by the Records Manager, as specified in the University of Sydney Recordkeeping Manual.

The study results will be used in a higher research degree project. Anonymous data will be stored on an online secure password protected research database accessed within the Institute of Musculoskeletal Health supported by the University of Sydney.

8. Storage of Data

Data will be stored securely within the University of Sydney's Research Data Store (RDS). The RDS is a secure, password protected, web-based, data management tool designed for research purposes. Data stored in the RDS is stored on servers in the University of Sydney data centre. Data is secured and regularly backed-up to protect privacy and confidentiality.

© 2025 Altinger G et al. *JAMA Network Open*

9. Future use of Data

The data collected in this project may also be used in future research studies. The results of this study and non-identified raw data may also be shared in the future with national and international collaborators. If any stored data are used for future research, the research will first be reviewed and approved by an appropriately constituted Ethics Committee.

10. Further Information

When you have read this information, and you have any questions feel free to reach out to Dr Adrian Traeger via Adrian.traeger@sydney.edu.au.

This information sheet is for you to keep.

11. Ethics Approval and Complaints

This study has been approved by the Ethics Review Committee (RPAH Zone) of the Sydney Local Health District. Any person with concerns or complaints about the conduct of this study should contact the Executive Officer on 02 9515 6766 or SLHD-RPAEthics@health.nsw.gov.au and quote protocol number X24-0060.

© 2025 Altinger G et al. *JAMA Network Open*

eTable 1. Scenario 1 – Surgery referral scenario and randomized treatment alternatives presented to control and intervention groups

Clinical Scenario – osteoarthritis<br>The patient is a 67-year-old male, trades person with chronic right hip pain. The diagnosis is osteoarthritis. He has been seeing a physiotherapist and walks daily. You have tried one nonsteroidal anti-inflammatory medication (ibuprofen 600mg three times daily with food) and have stopped them due to lack of efficacy. You decide to refer the patient to an orthopedic surgeon for consideration of hip replacement surgery. The patient agrees to this plan.
Control: Participants given one treatment alternative<br>Before the end of the consultation, however, you check the patients drug history and find that there is a covered nonsteroidal medication that this patient has not tried (see below).<br>What do you do? (please select one)<br>*There is no 'right' answer. Assume there is no difference in financial costs for any option.*<br>a. Refer to orthopedic surgeon and do not start any new medication.<br>b. [Alternative option randomly drawn from below list]<br>[1 alternative will be randomly inserted from the below options:<br>• Refer to orthopedic surgeon and also start indomethacin 25mg two times a day with food.<br>• Refer to orthopedic surgeon and also start Naproxen 500mg twice a day with food.<br>• Refer to orthopedic surgeon and also start diclofenac 50mg two times a day with food.]
Treatment: Participants given two treatment alternatives<br>Before the end of the consultation, however, you check the patients drug history and find that there are 2 covered nonsteroidal medications that this patient has not tried (see below)<br>What do you do? (please select one)<br>*There is no 'right' answer. Assume there is no difference in financial costs for any option.*<br>a. Refer to orthopedic surgeon and do not start any new medication.<br>b. [Alternative option randomly drawn from below list]<br>c. [Alternative option randomly drawn from below list]<br>[2 alternatives will be randomly inserted from the below options:<br>• Refer to orthopedic surgeon and also start indomethacin 25mg two times a day with food.<br>• Refer to orthopedic surgeon and also start naproxen 500mg twice a day with food.<br>• Refer to orthopedic surgeon and also start diclofenac 50mg two times a day with food.]

© 2025 Altinger G et al. *JAMA Network Open*

eTable 2. Scenario 2 – opioid prescribing scenario and randomized treatment alternatives presented to control and intervention groups

Clinical Scenario – opioid prescribing<br>The patient is a 41-year-old male, office worker who you've been seeing to help manage his chronic low back pain for a few months. He has no history of trauma or cancer, attends physiotherapy regularly and enjoys exercise (regular walking, occasional yoga and playing ball games with his kids) when he isn't in pain. Approximately 2 weeks ago he received a prescription for a 3-day supply of oxycodone 5mg, up to twice daily, to help him cope with flare ups, which he says he responded well to. He has presented for a refill of his prescription.
Control: Participants given one treatment alternatives<br>When initiating the order, you receive a pop-up notification on your computer suggesting that you consider an NSAID.<br>What do you do? (please select one)<br>*There is no 'right' answer. Assume there is no difference in financial costs for any option.*<br>a. Continue with prescription for another 3-day supply of oxycodone 5mg up to twice daily.<br>b. [Alternative option randomly drawn from below list].<br>[1 alternative will be randomly inserted from the below options:<br>• Prescribe indomethacin 25mg two to three times per day with food.<br>• Prescribe diclofenac 50mg two times a day with food.<br>• Prescribe naproxen 500mg two times a day with food.<br>• Prescribe ibuprofen 600mg three times a day with food.]
Intervention subgroup 1: Participants given two treatment alternatives<br>When initiating the order, you receive a pop-up notification on your computer suggesting you consider an NSAID.<br>What do you do? (please select one)<br>*There is no 'right' answer. Assume there is no difference in financial costs for any option.*<br>a. Continue with prescription for another 3-day supply of oxycodone 5mg PO up to twice daily.<br>b. [Alternative option randomly drawn from below list].<br>c. [Alternative option randomly drawn from below list].<br>[2 alternatives will be randomly insert from the below options:<br>• Prescribe indomethacin 25mg two to three times per day with food.<br>• Prescribe diclofenac 50mg two times a day with food.<br>• Prescribe naproxen 500mg two times a day with food.<br>• Prescribe ibuprofen 600mg three times a day with food.]
Intervention subgroup 2: Participants given three treatment alternatives<br>When initiating the order, you receive a pop-up notification on your computer suggesting you consider an NSAID.<br>What do you do? (please select one)

© 2025 Altinger G et al. *JAMA Network Open*

There is no 'right' answer. Assume there is no difference in financial costs for any option.<br>a. Continue with prescription for another 3-day supply of oxycodone 5mg up to twice daily.<br>b. [Alternative option randomly drawn from below list].<br>c. [Alternative option randomly drawn from below list].<br>d. [Alternative option randomly drawn from below list].<br>[3 alternatives will be randomly insert from the below options:<br>• Prescribe indomethacin 25mg two to three times per day with food.<br>• Prescribe diclofenac 50mg two times a day with food.<br>• Prescribe naproxen 500mg two times a day with food.<br>• Prescribe ibuprofen 600mg three times a day with food.]
Intervention subgroup 3: Participants given four treatment alternatives<br>When initiating the order, you receive a pop-up notification on your computer suggesting you consider and NSAID.<br>What do you do? (please select one)<br>*There is no 'right' answer. Assume there is no difference in financial costs for any option.*<br>a) Continue with prescription for another 3-day supply of oxycodone 5mg up to twice daily.<br>b) Prescribe indomethacin 25mg two to three times per day with food.<br>c) Prescribe diclofenac 50mg two times a day with food<br>d) Prescribe naproxen 500mg two times a day with food<br>e) Prescribe ibuprofen 600mg three times a day with food.

© 2025 Altinger G et al. *JAMA Network Open*

eMethods 2. GEE model

Primary Analysis: Interaction model with GEE estimation to account for cluster structure

Regression equation:

$$logit(P(Y=1))=\alpha\hat{}+\beta\hat{}1group+\ \beta\hat{}2S+\ \beta\hat{}3group*S \mathrm{logitPY}=1=\alpha\hat{}+\beta\hat{}1\mathrm{group}+\ \beta\hat{}2\mathrm{S}+\ \beta\hat{}3\mathrm{group}*\mathrm{S}$$

Where
Logit (p(y=1)) = log odds of choosing a high value alternative

$\alpha\ \hat{}\alpha\ \hat{}$ = intercept

$\beta\hat{}1\beta\hat{}1$ = effect of intervention in scenario 1

$\beta\hat{}2\ \beta\hat{}2$ = effect of scenario in control group

$\beta\hat{}3\ \beta\hat{}3$ = effect of interaction term (ie the interaction between intervention group and scenario)

© 2025 Altinger G et al. *JAMA Network Open*
