# Supplementary material for: Multiple Suggested Care Alternatives and Decision-Making of Primary Care Physicians: A Randomized Clinical Trial
Source: JAMA Netw Open. 2025 Nov 13;8(11):e2542949. doi: 10.1001/jamanetworkopen.2025.42949 (PMC12616461; doi:10.1001/jamanetworkopen.2025.42949)
Supplement: Supplement 2. — Trial Protocol [file jamanetwopen-e2542949-s002.pdf]

Research Protocol

**Protocol Number**	X24-0060
**Study Title**	Revisiting medical decision making in situations that offer multiple alternatives: a randomised experiment
**Coordinating Principal Investigator**	Dr Adrian Traeger, Sydney Local Health District, University of Sydney
**Signature: Adrian Traeger**	Date: 15.3.24
**Co-investigators**	N/A.
**Student Investigator(s) (if applicable)**	Gemma Altinger is conducting the study to partially fulfil the requirements of Master of Philosophy. Gemma will complete the analysis and drafting the results manuscript under the supervision of Adrian Traeger.

Ethics Statement:

The study will be conducted in accordance with the *National Statement on Ethical Conduct in Human Research* (2007) (Link to National Statement) , the *CPMP/ICH Note for Guidance on Good Clinical Practice* (Link to CPMP/ICH) and consistent with the principles that have their origin in the Declaration of Helsinki. Compliance with these standards provides assurance that the rights, safety and well-being of trial participants are respected.

BACKGROUND AND RATIONALE

Unwarranted healthcare variation is the inconsistency of clinical care that is not accounted for by patient symptoms or preferences and is broadly viewed as an indicator of healthcare quality.[1] At the clinician level, variation in medical opinion, lack of awareness of guidelines, uncertainty, and beliefs? have all been proposed to contribute to healthcare variation.[2] Despite efforts to reduce unwarranted healthcare variation through dissemination of guidelines, public awareness campaigns and professional development courses, variations in use of effective care and patient safety are considered significant problems internationally.[2,3]

The influence of cognitive biases could also help explain why unwarranted healthcare variation occurs.[4] Cognitive biases are cognitive processes that can lead to sub-optimal decision making. Despite expert knowledge and judgement, clinicians are not immune to biases that affect decision making and have been associated with guideline-discordant care and diagnostic error.[5-8] For example, status quo bias occurs when a decision maker maintains the current course, the previous decision or the default option, rather than changing course or choosing an alternative option.[9,10] In clinical decision making, status quo bias could look like a clinician prescribing the default quantity of a medication as presented in the electronic health system, rather than reducing or increasing the quantity based on patient need.

Normative economic theory suggests that having more choice increases the decision maker's ability to make a satisfactory decision. Under this assumption the experimental condition that presents two alternatives to the status quo, offers twice the number of reasons to switch from the status quo. However, according to literature on consumer decision-making, the more similar options that are introduced that are equal in attractiveness or trade-off complexity could increase the challenge in calculating the risks and benefits for each option, leading to "choice overload" and suboptimal decisions. A meta-analysis by Chernev et al. (2015) on consumer decision-making found that longer choice lists are more likely to lead to choice overload when the decision is difficult, the choice set is complex, and/or if the decision maker does not have strong prior preferences.[11] Chernev found deferral of choice or reduced choice switching as

strong measures of choice overload, both of which are characteristics of status quo bias. Although none of the studies were conducted on physician decision-making, status quo bias could be a contributing factor to unwarranted healthcare variation: clinical decision making is complex and often involves many treatment alternatives.[12]

Some studies have investigated the influence of the number of treatment alternatives on status quo bias. According to Redelmeier and Shafir (1995), the introduction of an additional care alternative made clinicians significantly more likely to choose the status quo option *(Status quo choice = 53% with 1 alternative vs 72% with 2 alternatives; P<.005)*. These findings failed to replicate in a later study by Roswarski (2006) *(54.5% with 1 alternative vs. 56.0% with 2 alternatives, P = 0.841)*.[13] While the premise of both experiments was sound, both studies are limited regarding external validity and internal validity. Both studies recruited academic physicians and focused on a single scenario which is now clinically out of date. Ideally vignette studies should present more than one scenario to account for within-physician correlations and to optimise external validity.[14]

Suggesting effective treatment alternatives, for example through computer alerts, is one approach to improving care and reducing unwarranted variation.[15] However, if the hypothesis that the number of treatment alternatives provided can increase the likelihood of status quo bias is true, such behavioural interventions could have unintended effects. A pop-up alert suggesting effective alternatives to medicine A (the status quo) could result in clinicians doing more of the status quo. For example, the upcoming NUDG-ED trial (ACTRN12623001000695) aims to reduce the use of opioids for patients with uncomplicated low back pain in the emergency department by using computer alerts that suggest NSAID alternatives. For an intervention like this, knowing if status quo bias can be triggered by introducing more choice alternatives would be useful information for intervention design. If status quo bias is triggered in a controlled survey environment, it is possible that the risk of this cognitive bias increases in real clinical contexts where choice options are larger and involve real risk and patient expectations.[16]

A study by Meeker et. al. (2016) that did involve real clinical decision making found that offering alternatives to inappropriate antibiotic prescribing for acute respiratory tract infections was smaller than expected effects.[17] The decision support alert offered 15 treatment alternatives. It is possible that offering fewer alternatives could have reduced the trade-off complexity and choice overload and led to more decision makers switching from the status quo option.

Alerts in electronic health ordering systems are gaining popularity as a method to improve clinical care. A study looking to identify the number of medical alerts triggered in a 726-bed academic medical center between November 2017 and June 2018 found that 1,625,341 interruptive alerts across 1,474 different categories were triggered.[18] Given the prevalence of medication alerts and decision support systems that are embedded in daily practice, more evidence on the impact of suggesting alternatives and ways of improving these systems is required.

AIMS

Our primary aim is to determine whether primary care physicians are more likely to experience status quo bias in clinical scenarios with multiple treatment alternatives compared with a single treatment alternative. Specifically, we will determine if offering 2 or more treatment alternatives increases the probability of the status quo option being chosen compared to 1 treatment alternative.

METHODS

Study Design

We will conduct a variation of the Redelmeier and Shafir (1995) experiment where we will update the scenario to be more clinically relevant to the current clinical care settings (e.g. medicines used 30 years ago are currently not recommended). We will also add an additional scenario to examine within- and between-clinician variation and increase the external validity of the study.

We will recruit 396 primary care physicians currently in clinical practice in the United States (U.S.). Primary care physicians will be recruited by the company Qualtrics and are blinded to the purpose of the study. As this study is exploring status quo bias anonymity is an important feature of this study design.

198 will be randomly assigned to the control group (one treatment alternative) and 198 to the intervention group (two or more treatment alternatives). Each participant will complete 2 clinical scenarios. Primary care physicians who complete the scenarios and survey will receive $35AUD equivalent compensation for their time. Compensation is administered by Qualtrics, through participants existing membership agreement. After completing the survey participants will have the option to provide their email if they would like to receive the results of the study when complete. This form will be stored separately and cannot be linked to participant survey responses. Participants do not need to provide their email via this survey in order to receive compensation from Qualtrics.

Participants will be randomly assigned to the control and intervention groups. The randomisation schedule will be computer generated by Qualtrics. Allocation will be concealed from participants and investigators. Staff at Qualtrics will be aware of group allocation being responsible for randomisation and data collection, however they are a third party who will not be involved in analysis. All investigators will be blinded to allocation until after analysis is complete.

Participants in the control group will receive 2 clinical scenarios that each include a common presentation in primary care. Within the scenarios a treatment option will be described as the status quo decision (e.g. a previous decision the participant had made or one that is considered the default option). The control group will receive 1 treatment alternative to the status quo.

Participants in the intervention group will receive the same 2 clinical scenarios as the control group, but instead of one treatment alternative they will be given two or more treatment alternatives to choose from. For scenario 2 the intervention group will be further randomised into one of three intervention subgroups. These intervention subgroups will present participants with either two, three or four treatment

alternatives. The order of the scenarios and the treatment alternatives will be randomised to reduce order effects and the influence of prescribing preferences. They will have approximately 2 minutes to complete each scenario and can select one choice only. Data will be collected via Qualtrics online survey.

Study Sites

This study will be coordinated by the Royal Prince Alfred Hospital. Participants of this online experiment will be recruited via Qualtrics. Participants will be eligible primary care physicians in the USA who have identified themselves as wanting to participate in online research. Data will be stored and analysed via the University of Sydney Research Data Store.

SURVEY/QUSTIONNAIRE

STUDY POPULATION

INCLUSION CRITERIA

We will recruit 396 primary care physicians currently in clinical practice in the United States (U.S.) that are enrolled with Qualtrics.

EXCLUSION CRITERIA

Academic physicians or members of the public. Participants that do not consent to partaking in this study.

RECRUITMENT AND CONSENT

Participants will be eligible primary care physicians in the USA who have identified themselves as wanting to participate in online research by enrolling in the Qualtrics participant registry. Primary care physicians will be recruited by the company Qualtrics. Qualtrics will provide 396 completed surveys from primary care physicians. Researchers will have no direct contact with participants.

Participant information will be presented, and consent will be obtained via the Qualitrics survey platform. Once consent is obtained, 198 will be randomly assigned to the control group (one treatment alternative) and 198 to the intervention group (two or more treatment alternatives). Each participant will complete 2 clinical scenarios. Participants who complete the scenarios and survey will receive $35AUD equivalent

compensation for their time. Qualtrics pays participants through their existing membership. Participants will be blinded to the purpose of the study.

Qualtrics are contracted to provide us with 396 completed participant surveys, so there is no risk in not meeting sample size required. Participants and their results will be anonymous collected via a third party so their relationship or any future relationship with researchers and the local health district will not be affected.

DATA COLLECTION

Participants will be randomly assigned to the control and intervention groups. The randomisation schedule will be computer generated by Qualtrics. Allocation will be concealed from participants and investigators. Staff at Qualtrics will be aware of group allocation being responsible for randomisation and data collection, however, will not be involved in analysis. At least 2 investigators will be blinded to allocation until after analysis is complete.

Participants in the control group will receive 2 clinical scenarios that each include a common presentation in primary care. Within the scenarios a treatment option will be described as the status quo decision (e.g. a previous decision the participant had made or one that is considered the default option). The control group will receive 1 treatment alternative to the status quo.

Participants in the intervention group will receive the same 2 clinical scenarios as the control group, but instead of one treatment alternative they will be given two or more treatment alternatives to choose from. For scenario 2 the intervention group will be further randomised into one of three intervention subgroups. These intervention subgroups will present participants with either two, three or four treatment alternatives.

The order of the scenarios and the treatment alternatives will be randomised to reduce order effects and the influence of prescribing preferences. Surveys will be multiple choice questions; no free text will be collected. They will have approximately 2 minutes to complete each scenario and can select one choice only.

DATA ANALYSIS

The primary outcome is the proportion of primary care physicians choosing the status quo option in a choice set. The hypothesis is that exposing participants to 2 or more equivalent treatment choice alternatives in an order set increases the likelihood of choosing the status quo option compared with one treatment alternative.

A generalised linear mixed model will be used to test significance for main effects of exposure to two or more treatment alternatives on the proportion choosing the status quo option, across the two scenarios. For this analysis, the Scenario 2 intervention subgroups will be considered as one group.

<u>Potential effect modifiers</u>

Clinician experience through years of clinical practice and if they supervise medical students will be being collected as potential moderators. Roswarski (2006) found that increased clinician experience and student supervision reduced the likelihood of the status quo option being chosen when a choice set is expanded to include more treatment alternatives.

We will conduct an exploratory subgroup analysis to identify whether any effects of multiple treatment alternatives on status quo bias increases with exposure to 2, 3 or 4 treatment alternatives. A sensitivity analysis will be performed to check if the results were robust to variation in participant attention.

WITHDRAWAL OF CONSENT

Participants can withdraw at any time by closing the online survey. However, only finished surveys will be eligible for financial reimbursement. Data collected up until the time they withdraw may be included in the study. The information you provide for this study will be anonymous. This means that it cannot be

re-identified once participants have submitted the survey, therefore their information cannot be withdrawn from the study after they submit the survey.

DATA STORAGE AND ARCHIVING/RETENTION

Storage:

Data will be stored securely within the University of Sydney's Research Data Store (RDS). The RDS is a secure, password protected, web-based, data management tool designed for research purposes. Data stored in the RDS is stored on servers in the University of Sydney data centre. Data is secured and regularly backed-up to protect privacy and confidentiality. The files will be retained for 10 years from the day the study is completed. Once the retention expires the files will be disposed of. Data will be securely deleted from the RDS once appropriate approval is given by the Records Manager, as specified in the University of Sydney Recordkeeping Manual.

FUTURE USE OF DATA

The data collected in this project may also be used in future research studies. The results of this study and non-identified raw data may also be shared in the future with national and international collaborators. If any stored data are used for future research, the research will first be reviewed and approved by an appropriately constituted Ethics Committee.

RESEARCH DATA MANAGEMENT PLAN (RDMP)

We have completed a research data management plan and attached this to the application in REGIS.

PRIVACY AND CONFIDENTIALITY

All the information collected for the study is anonymous and will be treated confidentially. Data will be stored on a research database at the University of Sydney. Emails collected for distribution of study results will be stored separately and cannot be connected to participant responses. A Research Data Management Plan (RDMP) has been created using the Sydney Local Health District RDMP tool.

ETHICS AND PROTOCOL AMENDMENTS

The conduct of this study will commence once the initial approval process has been completed through Ethics and Governance authorisation at the Royal Prince Alfred.

CONFLICTS OF INTEREST AND MANAGEMENT PLAN

No conflicts of interest have been identified.

PUBLICATION POLICY

AT and GA have both been involved in the conception and design of this study. All authors of the results manuscript will be required to meet the Australian Code for the Responsible Conduct of Research Authorship criteria. Authors will need to make significant intellectual or scholarly contribution to the study and agree to be listed as an author.

Study participants can nominate if they would like to receive a copy of the study results after completing the survey.

STUDY TIMELINE

Task	Start Date	End Date
**Ethics Submission**	15 March 2024	15 March 2024
**Ethics Review and Approval**	10 April 2024	11 April 2024
**Advertising, recruitment and survey completion**	12 April 2024	30 April 2024
**Analysis of Data**	1 May 2024	1 June 2024
**Manuscripts Drafted**	2 June 2024	20 June 2024
**Submission of Publications and Final Reports**	21 June 2024	30 Jun 2024

REFERENCES

1. Duggan A, Koff E, Marshall V. Clinical variation: why it matters. *Medical Journal of Australia*. 2016;205(10):3-4.
2. Atsma F, Elwyn G, Westert G. Understanding unwarranted variation in clinical practice: a focus on network effects, reflective medicine and learning health systems. *International Journal for Quality in Health Care*. Jun 4 2020;32(4):271-274. doi:10.1093/intqhc/mzaa023
3. Hoffman JR, Cooper RJ. Overdiagnosis of disease: a modern epidemic. *Archives of Internal Medicine*. 2012;172(15):1123-1124. doi:10.1001/archinternmed.2012.3319
4. Oakes AH, Radomski TR. Reducing Low-Value Care and Improving Health Care Value. *JAMA*. 2021;325(17):1715-1716. doi:10.1001/jama.2021.3308
5. Gigerenzer G. Why Heuristics Work. *Perspectives on Psychological Science*. 2008/01/01 2008;3(1):20-29. doi:10.1111/j.1745-6916.2008.00058.x
6. Saini V, Garcia-Armesto S, Klemperer D, et al. Drivers of poor medical care. *The Lancet* 2017;390(10090):178-190. doi:10.1016/S0140-6736(16)30947-3
7. Crowley RS, Legowski E, Medvedeva O, et al. Automated detection of heuristics and biases among pathologists in a computer-based system. *Advances in Health Sciences Education*. 2013;18(3):343-363. doi:10.1007/s10459-012-9374-z
8. Blumenthal-Barby JS, Krieger H. Cognitive Biases and Heuristics in Medical Decision Making: A Critical Review Using a Systematic Search Strategy. *Medical Decision Making*. 2015;35(4):539-557. doi:10.1177/0272989X14547740
9. Suri G, Sheppes G, Schwartz C, Gross JJ. Patient Inertia and the Status Quo Bias: When an Inferior Option Is Preferred. *Psychological Science*. 2013;24(9):1763-1769. doi:10.1177/0956797613479976
10. Zeckhauser R, Samuelson W. Status Quo Bias in Decision-Making. *Journal of Risk and Uncertainty*. 02/01 1988;1:7-59. doi:10.1007/BF00055564
11. Chernev A, Bockenholt U, Goodman J. Choice Overload: A Conceptual Review and Meta-Analysis. *Journal of Consumer Psychology*. 04/01 2015;25:Pages 333–358. doi:10.1016/j.jcps.2014.08.002
12. Dean M, Kıbrıs Ö, Masatlioglu Y. Limited attention and status quo bias. *Journal of Economic Theory*. 2017;169:93-127. doi:10.1016/j.jet.2017.01.009
13. Roswarski TE, Murray MD. Supervision of Students May Protect Academic Physicians from Cognitive Bias: A Study of Decision Making and Multiple Treatment Alternatives in Medicine. *Medical Decision Making*. 2006;26(2):154-161. doi:10.1177/0272989X06286483
14. Sheringham J, Kuhn I, Burt J. The use of experimental vignette studies to identify drivers of variations in the delivery of health care: a scoping review. *BMC Medical Research Methodology*. 2021/04/22 2021;21(1):81. doi:10.1186/s12874-021-01247-4
15. Kharel P, Zadro JR, Ferreira G, et al. Can language enhance physical therapists' willingness to follow Choosing Wisely recommendations? A best-worst scaling study. *Brazilian Journal of Physical Therapy*. 2023/07/01/ 2023;27(4):100534. doi:https://doi.org/10.1016/j.bjpt.2023.100534
16. Linder JA, Doctor JN, Friedberg MW, et al. Time of day and the decision to prescribe antibiotics. *JAMA internal medicine*. Dec 2014;174(12):2029-31. doi:10.1001/jamainternmed.2014.5225
17. Meeker D, Linder JA, Fox CR, et al. Effect of Behavioral Interventions on Inappropriate Antibiotic Prescribing Among Primary Care Practices: A Randomized Clinical Trial. *JAMA*. 2016;315(6):562-70. doi:10.1001/jama.2016.0275
18. Chien SC, Chin YH, Yoon CH, et al. A novel method to retrieve alerts from a homegrown Computerized Physician Order Entry (CPOE) system of an academic medical center: Comprehensive alert characteristic analysis. *PLoS One*. 2021;16(2):e0246597. doi:10.1371/journal.pone.0246597
19. Redelmeier DA, Koehler DJ, Liberman V, Tversky A. Probability Judgment in Medicine: Discounting Unspecified Possibilities. *Medical Decision Making*. 1995/08/01 1995;15(3):227-230. doi:10.1177/0272989X9501500305

20. Hoffmann TC, Glasziou PP, Boutron I, et al. Better reporting of interventions: template for intervention description and replication (TIDieR) checklist and guide. *BMJ*. 2014;348:g1687. doi:10.1136/bmj.g1687
21. NICE. Osteoarthritis in over 16s: diagnosis and management. NICE guideline [NG226]. 2022.
22. Care ACoSaQiH. Low back pain clinical care standard. 2022.
