# Supplementary material for: Multiple Suggested Care Alternatives and Decision-Making of Primary Care Physicians: A Randomized Clinical Trial
Source: JAMA Netw Open. 2025 Nov 13;8(11):e2542949. doi: 10.1001/jamanetworkopen.2025.42949 (PMC12616461; doi:10.1001/jamanetworkopen.2025.42949)
Supplement: Supplement 3. — Data Sharing Statement [file jamanetwopen-e2542949-s003.pdf]

Data Sharing Statement

Altinger. Multiple Suggested Care Alternatives and Decision-Making of Primary Care Physicians. *JAMA Netw Open*. Published November 13, 2025.
doi:10.1001/jamanetworkopen.2025.42949

Data
Additional Information: Australian New Zealand Clinical Trials Registry, https://www.anzctr.org.au, ACTRN12625001025426
Data available: Yes
Data types: Deidentified participant data
How to access data: The data that support the findings of this study are available from the corresponding author, GA, upon request with ethics approval.
When available: With publication

Supporting Documents
Document types: None

Additional Information
Who can access the data: Researchers whose proposed use of the data has been approved by an ethics committee
Types of analyses: For any purpose or for a specified purpose
Mechanisms of data availability: With a signed data access agreement
